# Effects of *Acacia cyanophylla*-condensed tannin supply on growth performance, carcass characteristics and meat quality of fat-tailed Barbarine lambs studied under grazing conditions or in a feedlot

**DOI:** 10.5194/aab-69-495-2026

**Published:** 2026-09-03

**Authors:** Yathreb Yagoubi, Naziha Atti, Samir Smeti, Jihene Abidi, Mokhtar Mahouachi

**Affiliations:** 1 University of Carthage, Laboratoire des Production Animale et Fourragère, Institut National de la Recherche Agronomique de Tunisie, Rue Hedi Karray, Ariana 2049, Tunisia; 2 University of Jendouba, Laboratoire Appui à la Durabilité des Systèmes de Production Agricoles du Nord-Ouest, Ecole Supérieure d'Agriculture du Kef, Le Kef 7119, Tunisia

## Abstract

The aim of this study is to evaluate the effects of dietary supplementation with acacia foliage, a tannin source, and feeding systems on Barbarine lambs' growth, carcass traits, and meat quality under grazing and feedlot conditions. Twenty-eight male lambs (5-month-old, 22.5 ± 3.6 kg) were divided into four groups based on body weight (BW) and assigned to one of four diets for a 60 d trial. Two groups were raised in stalls (S) and fed hay and concentrate, while two grazed on pasture (P) with the same concentrate supplementation. Within each system, one group received 100 g of *Acacia cyanophylla* daily (S-T, P-T), and the other did not (S-0T, P-0T). Pasture-fed lambs had higher total daily intake, average daily gain (ADG) and slaughter body weight than stall-fed lambs. Carcass weights and dressing percentages were also higher for grazing lambs. Tannin supplementation did not affect carcass traits or meat quality. Grazing lambs showed more developed red organs and higher fat deposition. Meat quality, including pH, color, chemical composition and sensory attributes, was similar across groups. Low doses of dietary tannins improved growth and dressing percentages when combined with a low-tannin diet but reduced growth rates when basal diets were tannin-rich. These findings suggest that dietary tannins' effects depend on the basal diet's composition.

## 1 Introduction

Currently, consumers are increasingly demanding more natural and safer foods of animal origin with a distinctive flavor and nutrient substances that are important for health. Several factors could affect animal growth and the meat quality, such as feeding system and diet nature (Hajji et al., 2016; Renna et al., 2019). To promote animals' performance, some antibiotic growth promoters have been excessively used for several decades in ruminants' and broilers' diets to promote growth and to prevent diseases (Huang et al., 2018; García-Salas et al., 2022). However, the accumulation of toxic residues in meat due to the inappropriate use of these products could affect consumer health (Mund et al., 2017). For this reason, these compounds were recently prohibited (Valenzuela-Grijalva et al., 2017), and great interest has arisen to search alternative natural products such as additives to improve the productive response of animals (Salami et al., 2018). The use of secondary metabolites such as tannins, which are polyphenolic compounds derived from plants, is highlighted. These have received special attention and are among the most studied bioactive compounds, particularly in ruminants (Huang et al., 2018; Tontini et al., 2021). They have been studied in animal nutrition and production, especially their biological activities as they act as antioxidants and antimicrobials and have anti-inflammatory properties (Huang et al., 2018). However, the results have been variable, depending on the source of the tannins and the dose used in the ration. While a high dose (2.5 %) of condensed tannins resulted in a decrease in lamb performance, a small level (1.5 %) in diets could improve or not affect lamb growth and meat quality (Guerreiro et al., 2020). Tannins could play an important role in the nutritional value of the feed, the quality of the obtained products, and the animals' welfare and health (Jerónimo et al., 2016). Among several others, the *Acacia cyanophylla* Lindl is a tannin-rich legume (60 g kg^−1^ dry matter) that is widespread in Tunisia. The use of small amounts of *Acacia cyanophylla* Lindl foliage rich in tannins can naturally play that role (Ben Salem et al., 2005) and allow a better flow of amino acids and normally less fatty carcasses.

On the other hand, the feeding systems are widely acknowledged to significantly affect the growth, development, body health of livestock and meat characteristics (Priolo et al., 2001; Zhao et al., 2023). The meat issued from ruminants studied under grazing conditions has different characteristics from those of animals studied in a feedlot and fed hay and concentrate, in terms of color, flavor and fatty acid composition (Priolo et al., 2001; Hajji et al., 2016). Furthermore, we have seen marked changes in consumer lifestyles and consumption concepts through the increasing demand for clear information regarding the animals' feeds and for high food quality, low in fat but rich in healthy polyunsaturated fatty acids. For instance, meat and milk composition has important repercussions on human health. Consumers generally preferred meat issued from grazing and considered it natural, healthy and respectful of animal welfare (Prache et al., 2005; Smeti et al., 2014).

Therefore, it is important to differentiate between meat from lambs reared on pasture or kept in a feedlot throughout the carcass characteristics. In the same context, grazing on pasture has been recognized as a cost-effective method for lamb production, providing a healthy and green meat that is highly valued by consumers (Carrasco et al., 2009). In addition, grazing lambs reached slaughter age (6 months) with a higher weight and less fat than feedlot lambs fed on hay and concentrate (Atti and Abdouli, 2001).

Then, the aim of this work was to study the effect of grazing cultivated pasture (vetch) or feedlot conditions associated with acacia supply in small quantities as a tannin source on growth performance, carcass traits and meat quality of fat-tailed Barbarine lambs.

## 2 Material and methods

This trial was carried out at Lafareg, Beja, the experimental farm of the National Institute of Agronomic Researches of Tunisia (INRAT). Lafareg belongs to the sub-humid bioclimatic stage (36^′^73° N, 9^′^18° E). The average annual precipitation and humidity are 558 mm and 63.5 %, respectively. Animal care, management procedures, transport and slaughtering meet ethical guidelines and adhere to Tunisian legal requirements (The Livestock Law No. 2005-95 of 18 October 2005, Chapter II; Sects. 1 and 2 relative to the slaughter of animals).

### 2.1 Animals, diets and experimental design

The experiment began on 30 March and lasted 60 d. Twenty-eight male fat-tailed Barbarine lambs (5-month-old, 22.5 ± 3.6 kg) were divided into four homogeneous groups of seven lambs each according to their body weight (BW). Before starting the experiment, all animals were treated against internal and external parasites and enterotoxemia. Then, lambs were randomly allocated to one of four diets. Two groups were raised in stalls (S) and fed 400 g of concentrate and hay ad libitum, while the two other groups were managed in a rotational grazing system on a vetch-cultivated pasture (P) and also supplemented with 400 g d^−1^ of concentrate. The vetch pasture was divided into four paddocks. Herbage mass was determined before entering each paddock by cutting 10 quadrates (0.25 m^2^ per quadrat), and the whole grass production was calculated according to this sample weight and the paddock area. Furthermore, 10 % of forage production is considered wasted, and only 90 % of this production was ingested by lambs. For each feeding system, only one group received 100 g per head per day of acacia as a tannin source (S-T and P-T), and the two other groups did not receive it (S-0T and P-0T). The acacia was distributed 1 h prior to the distribution of other feeds. The feeding trial lasted 77 d, during which experimental diets were offered twice a day. All lambs had free access to water. Refusals were weighed before the feed distribution, and representative samples were taken for laboratory analysis. Individual feed intakes were measured daily. Lambs were weighed at the beginning of the study and then weekly before the morning meal. Lambs' average daily gain (ADG) was calculated by the difference between the final and the initial weights divided by the total number of days.

### 2.2 Slaughter procedure, measurements, carcass cutting and dissection

At the end of the experimental feeding period, all lambs were slaughtered in the abattoir of the INRAT to evaluate carcass characteristics and meat quality. They were fasted 12 h before slaughter with only access to water. Slaughter body weight (SBW) was recorded immediately before slaughter. Then, non-carcass components such as skin, head, feet, gastro-intestinal tract, red organs (heart, liver, lungs and trachea) and internal fats (omental and mesenteric) were removed and weighed. All fractions of the digestive tract (reticulo-rumen + omasum (rumen), abomasum and intestine) were weighed full and then empty after hand rinsing, in order to determine the weight of the digestive contents. Empty body weight (EBW) was calculated as the difference between SBW and the weight of digestive contents. Hot carcass weight (HCW) was recorded, and then carcasses were stored at 4 °C. Cold carcass weight (CCW) was recorded 24 h postmortem after chilling at 4 °C.

Commercial (CDP) and real dressing percentage (RDP) were calculated according to the following formulae:

1CDP(%)=100×HCW/SBW,2RDP(%)=100×CCW/EBW.

The kidneys, kidney fat, testis and fat tail were removed and weighed. Once the tail was removed, each carcass was carefully halved longitudinally into sides. The left side was sectioned into six standardized commercial joints (Leg, shoulder, neck, ribs, loin and breast) according to Colomer-Rocher et al. (1988). Each joint was weighed and dissected into muscle, bone, fat (pelvic, subcutaneous, intermuscular) and waste (tendons, connective tissue etc.) to estimate the tissular composition.

### 2.3 Meat physico-chemical properties

All meat attributes were measured on the *longissimus dorsi* muscle. Meat color parameters were determined according to the Commission Internationale de l'Eclairage (CIE, 1986) using a Minolta CR-400 chromameter directly on the muscle surface, where the colorimetric indices as lightness ( L* ), redness ( a* ) and yellowness ( b* ) were recorded. The pH was measured immediately 1 h after slaughter and after 24 h using a penetrating electrode connected to a portable pH meter (HI 99163; Hanna Instruments, Romania) after calibration with two buffers (7.00 and 4.01). To determine water cooking loss (WCL) according to Boccard et al. (1981), meat samples were initially weighed ( $W_{i}$ ) and held in plastic bags and then immersed in a water bath at 75 °C and heated for 30 min until the internal temperature reached 75 °C. Then, the bags were cooled and blotted dry with paper towels. The cooked meat was weighed again (final weight, $W_{f}$ ). The WCL was calculated as 100× ( $W_{i}-W_{i})/W_{i}$ . For the chemical composition, meat samples were lyophilized (DM) and ground (1 mm screen). Ash was determined by combustion at 600 °C for 8 h. Nitrogen (N) was determined by the Kjeldahl method, and then the proteins were calculated as N × 6.25. Meat intramuscular fat was determined according to the method described by the AOAC (1997).

### 2.4 Sensory analysis

For sensory analysis, unsalted samples of Longissimus thoracis and lumborum (LTL) were roasted in aluminum paper in a pre-heated oven at 180 °C for 40 min. Each sample was cut into 10 pieces of 1 cm × 1 cm, and each piece was coded and served randomly for testing by a trained panel of 10 members. Each consumer was asked to evaluate 28 samples for tenderness (scale 1–9; 1 = extremely tough, 9 = extremely tender), juiciness (scale 1–9; 1 = extremely dry, 9 = extremely juicy), flavor (scale 1–9; 1 = very poor, 9 = very good) and overall acceptability (scale 1–9; 1 = not acceptable, 9 = extremely acceptable). Bread and water were provided for panelists to refresh their palates between samples.

### 2.5 Feed analyses

The chemical composition of the feeds is shown in Table 1. Samples of hay, concentrate, acacia and vetch were dried (75 °C); ground (1 mm screen); and analyzed for DM (105 °C until constant weight), nitrogen (Kjeldahl method) and ash according to the Association of Official Analytical Chemists (AOAC) (1990). Neutral detergent fiber, acid detergent fiber and acid detergent lignin were analyzed using an ANKOM220 fiber analyzer (ANKOM Technology Corp., Macedon, NY, USA).

**Table 1:** Chemical composition of the experimental diets (% DM basis).

Item	Vetch	Hay	Acacia	Concentrate
	grass			
Dry matter (%)	22	93	95	88
Organic matter	93.4	92.8	30	96.7
Crude protein	21.9	6.7	12.6	16.2
NDF	41.3	60.5	54.6	29
ADF	19.5	36.7	53.4	18.7
ADL	10.2	5.5	44.2	10.4

NDF: neutral detergent fiber; ADF: acid detergent fiber; ADL: acid detergent lignin.

### 2.6 Statistical analysis

Lambs' growth, carcass and non-carcass traits, regional and tissular composition, meat physico-chemical properties, and sensory attributes were analyzed using the general linear model (GLM) procedure of SAS (2004) in a 2×2 factorial design (feeding systems (pasture, P, and stall, S) and the presence or not of tannin supply (supplied, T, and non-supplied, 0T). The differences between groups were compared with Duncan's multiple range test (DMRT). The significance was declared to be p<0.05 .

## 3 Results

### 3.1 Feed intake and lambs' growth

The feed chemical composition is presented in Table 1. The percentages of crude protein are 21.9 %, 12.6 %, 16.2 % and 6.7 % for the grass, the acacia, the concentrate and the hay, respectively. The grass is richer in nitrogen than the hay (21.97 % DM vs. 6.7 % DM), which allows grazing lambs to benefit from this protein level.

The total dry matter intake was 839, 960, 1342 and 1442 g DM per head per day for S-0T, S-T, P-0T and P-T, respectively (Table 2). The proportions of forage intake are higher for the P group than those for feedlot animals (70 % vs. 53 %, respectively). However, the concentrate proportions were higher in the S groups (47 %) than the P ones (30 %). Then, the average daily consumption of forage for animals in pasture is estimated to 974 g of vetch per animal per day vs. 477 g of hay per animal per day). The lambs' ADG is shown in Table 3. The feeding system significantly affected the lambs' growth, with higher ADG for the P groups (180 g d^−1^) than that for the stall groups (141 g d^−1^). The tannin supply tended to increase the ADG for the stall groups and to decrease it for the P groups without a significant difference. Hence the lowest ADG (110 g) was recorded for group S-0T and the highest value for group P-0T (192 g). However, the interaction was significant ( p<0.01 ).

**Table 2:** Daily dry matter intake (g DM per head per day), forage-to-concentrate ratio and acacia tannin supplementation according to the feeding system.

Item	Daily intake	Forage	Concentrate	Acacia
	(g)	(%)	(%)	
P-0T	1342	70	30	–
P-T	1442	70	30	+
S-0T	839	52	48	–
S-T	960	54	46	+

P-0T: pasture without tannin supply; P-T: pasture with tannin supply; S-0T: stall feeding without tannin supply; S-T: stall feeding with tannin supply.

### 3.2 Carcass weights, dressing percentages and non-carcass components

Slaughter BW, empty BW, carcass weights, dressing percentages and weight of non-carcass components are summarized in Table 3. The majority of these parameters were affected by the feeding system but not by tannin supply. SBW and the RDP were significantly higher for both P groups and S-T; however, the EBW, as well as both hot and cold carcass weights and the commercial dressing percentage, was significantly higher for grazing lambs than stall ones. The red organs and kidney weights were more developed for grazing vetch lambs. The gut weights were similar for all groups ( P>0.05 ); however, the testis weight was significantly higher for both P groups and S-T compared to that of S-0T.

**Table 3:** Growth, carcass and non-carcass traits of lambs studied under grazing or in a feedlot and supplemented with condensed tannins from *Acacia cyanophylla*.

Item	P-0T	P-T	S-0T	S-T	SEM	P-FS	P-S	P-FS * S
Initial BW	21.9	22.2	20.7	23.6	0.62	0.5	0.15	0.9
Final BW	33.4^a^	32.2^ab^	27.3^b^	33.9^a^	0.82	0.18	0.19	0.04
ADG (g)	192^a^	167^ab^	110^b^	171^ab^	6.7	0.01	0.23	0.008
SBW (kg)	33.4^a^	32.2^ab^	27.3^b^	33.9^a^	0.82	0.18	0.19	0.04
EBW (kg)	27.7^a^	27.2^a^	21.1^b^	24.9^ab^	0.75	0.01	0.32	0.19
HCW (kg)	15.3^a^	14.9^a^	11.0^b^	13.9^ab^	0.47	0.01	0.23	0.12
CCW (kg)	14.7^a^	14.2^a^	10.6^b^	13.3^ab^	0.43	0.01	0.26	0.11
CDP (%)	45.6^a^	45.9^a^	40.2^b^	40.8^b^	0.51	0.001	0.68	0.90
RDP (%)	52.9^a^	52.3^a^	50.4^b^	53.5^a^	0.33	0.38	0.11	0.01
Gut (kg)	2.27	2.10	1.99	2.29	49.3	0.71	0.58	0.04
Red organs (kg)	1.30^a^	1.21^a^	1.07^b^	1.11^b^	24.8	0.007	0.63	0.21
Kidney (g)	48^a^	47^a^	38^b^	38^b^	1.16	0.001	0.85	0.78
Testis (g)	83^a^	92^a^	54^b^	93^a^	7.5	0.41	0.15	0.36

P-0T: pasture without tannin supply; P-T: pasture with tannin supply; S-0T: stall feeding without tannin supply; S-T: stall feeding with tannin supply; P-FS: P value of feeding system; P-S: P value of supply of tannin; P-FS * S: interaction between feeding system and supply of tannins; ADG: average daily gain; SBW: slaughter body weight; EBW: empty body weight; HCW: hot carcass weight; CCW: cold carcass weight; CDP: commercial dressing percentage; RDP: real dressing percentage. ^a,b^ Different letters within the same row (different dieats) differ significantly ( P<0.05 ).

### 3.3 Carcass joints' weights and proportions in the tailed carcasses and tissular composition

The majority of carcass joints (leg, shoulder, loin, breast and tail) were affected by the feeding system; however, the ribs and the neck were unaffected (Table 4). The feeding system also significantly affected the proportions of ribs, loin, neck and tail. Conversely, the dietary supplementation by tannins affect neither the carcass joints' weights nor their proportions in the tailed carcass. The proportions of the different carcass tissues are reported in Table 4. The highest muscle proportion was recorded for group S-0T compared to the rest of the groups; however a higher fat proportion was observed for both P groups and S-T.

**Table 4:** Carcass joints' weights (kg) and proportions in the tailed carcasses and tissular composition of lambs studied under grazing or in a feedlot and supplemented with condensed tannins from *Acacia cyanophylla*.

Item	P-0T	P-T	S-0T	S-T	SEM	P-FS	P-S	P-FS * S
Leg	2.48^a^	2.45^a^	1.88^b^	1.93^b^	72	0.001	0.97	0.80
Shoulder	1.45^a^	1.46^a^	1.15^b^	1.16^b^	47.4	0.009	0.9	0.96
Ribs	0.49	0.44	0.45	0.49	23.3	0.96	0.99	0.39
Loin	1.30^a^	1.20^a^	0.81^b^	0.99^b^	61	0.01	0.78	0.29
Breast	0.78^a^	0.73^a^	0.51^b^	0.57^b^	24.7	0.001	0.93	0.32
Neck	0.49	0.47	0.48	0.40	19.2	0.31	0.31	0.49
Tail	1.03^a^	0.87^b^	0.47^c^	0.76^b^	59.1	0.01	0.63	0.09
% in tailed carcass								
Leg	33.8	34.8	34.2	34.8	0.32	0.78	0.27	0.80
Shoulder	19.8	20.7	20.8	20.8	0.15	0.12	0.21	0.19
Ribs	6.6^b^	6.2^b^	7.9^a^	7.3^a^	0.21	0.01	0.30	0.87
Loin	17.7^a^	16.5^a^	14.5^b^	15.0^b^	0.37	0.01	0.64	0.33
Breast	10.7	10.3	9.3	10.1	2.06	0.08	0.66	0.24
Neck	6.7^b^	6.8^b^	8.6^a^	7.3^b^	0.14	0.001	0.10	0.05
Tail	6.5^a^	5.6^ab^	4.0^b^	5.4^ab^	0.24	0.02	0.63	0.04
Tissular composition								
Muscle (%)	54.9	56.7	58.8	55.7	0.55	0.23	0.61	0.05
Fat (%)	22.5^a^	21.0^a^	15.7^b^	20.4^a^	0.63	0.01	0.26	0.03

P-0T: pasture without tannin supply; P-T: pasture with tannin supply; S-0T: stall feeding without tannin supply; S-T: stall feeding with tannin supply; P-FS: P value of feeding system; P-S: P value of supply of tannin; P-FS * S: interaction between feeding system and supply of tannins. ^a,b,c^ Different letters within the same row (different diets) differ significantly ( P<0.05 ).

### 3.4 Meat quality

The meat physico-chemical properties are presented in Table 5. Initial and ultimate pH values were similar for all lambs. Water cooking loss and all color parameters (lightness ( L* ), redness ( a* ) and yellowness ( b* )) did not differ among groups ( P>0.05 ). The meat chemical composition (DM, ash, OM, protein and fat) was not affected by the dietary treatments ( P>0.05 ).

**Table 5:** Meat physico-chemical properties and sensory characteristics of lambs studied under grazing or in a feedlot and supplemented with condensed tannins from *Acacia cyanophylla*.

Item	P-0T	P-T	S-0T	S-T	SEM	P-FS	P-S	P-FS * S
Initial pH	6.8	6.9	6.8	6.7	0.16	0.11	0.62	0.36
Ultimate pH	5.9	5.8	5.8	5.7	0.15	0.06	0.42	0.84
Water cooking loss	16.6	14.3	16.6	13.4	3.66	0.25	0.49	0.28
Lightness ( L* )	43.5	40.9	44.3	45.4	3.90	0.78	0.48	0.14
Redness ( a* )	13.7	16	14.6	14.2	1.78	0.35	0.54	0.005
Yellowness ( b* )	7.0	7.4	9.1	8.6	1.36	0.18	0.43	0.40
Dry matter (%)	25	24.8	24.5	25.6	0.88	0.53	0.24	0.25
Ash (%)	6.0	4.7	5.5	4.5	1.71	0.26	0.30	0.23
Organic matter (%)	94	95.3	94.6	95.6	1.71	0.33	0.32	0.32
Protein	83.5	86.8	85.7	84.1	4	0.16	0.77	0.92
Fat	10.5	8.5	8.8	11.4	4.52	0.26	0.26	0.25
Tenderness	6.0	5.7	5.9	6.0	1.78	0.87	0.55	0.73
Juiciness	5.7	5.1	5.8	5.6	1.81	0.14	0.41	0.57
Flavor	6.7	6.3	6.8	6.2	1.55	0.45	0.20	0.60
Overall impression	5.9	5.5	6	5.6	1.74	0.71	0.05	0.56

P-0T: pasture without tannin supply; P-T: pasture with tannin supply; S-0T: stall feeding without tannin supply; S-T: stall feeding with tannin supply; P-FS: P value of feeding system; P-S: P value of supply of tannin; P-FS * S: interaction between feeding system and supply of tannins.

### 3.5 Meat sensory evaluation

The results of meat sensory analyses are summarized in Table 5. Overall, the sensorial quality was unaffected by the feeding system and the tannin supplement ( p>0.05 ), and the meat of all groups was judged averagely tender (5.7–6) and juicy (5.1–5.8), without a specific flavor, and was accepted by all the panelists.

## 4 Discussion

### 4.1 Lambs' growth performance

The results of the current study are consistent with those of Nuernberg et al. (2008), who found a higher average daily gain for grazing lambs on grass (127 g d^−1^) compared with those in stalls (113 g d^−1^). Similar results were recorded for lambs grazing on natural pastures and supplemented with 150–300 g of concentrate, which exhibited a 32.5 % increase in ADG compared to indoor-fed counterparts (Wang et al., 2015). Atti and Abdouli (2001) also found that raising Barbarine lambs on pasture and receiving the same concentrate amount as those in stalls recorded higher growth performance ( p<0.001 ). In contrast, it has been shown that this feeding system results in a 13.8 % reduction in final body weight (FBW) and a 28.7 % decrease in average daily gain (ADG) compared to indoor-fed lambs (Gabryszuk et al., 2014). The current result could be explained by the higher vetch intake for P groups (942 g DM per head per day) compared to that for S ones (439 and 460 g DM per head per day for groups S-0T and S-T, respectively) and the higher protein level in grass (22 %) compared to that of hay (6.7 %). Conversely, Majdoub-Mathlouthi et al. (2015) found no difference in ADG among lambs reared indoors and those reared on pastures. The high ADG for grazing lambs could be related to the higher DM intake and mostly to the high crude protein (CP) content in the diet (Yagoubi et al., 2018). This is consistent with the results of Titi et al. (2000), who found that increasing the level of protein in the diet leads to a higher average daily gain for Awassi lambs, going from 99 to 171 and 208 g for protein level rations of 12 %, 14 % and 16 %. However, other authors reported higher growth for animals raised in stalls compared to those on pasture (Ji et al., 2024). Contrary to our results, many results have reported that lower forage–concentrate ratios improved the performance compared to diets that had higher forage levels in dairy cows, goats and lambs (Chen et al., 2015; Jiang et al., 2022). These findings might be related to a greater nutrient intake, such as rapidly fermentable carbohydrates and quality protein, and higher nutrient digestibility found in high-concentrate diets compared to low-concentrate diets (Cantalapiedra-Hijar et al., 2014).

Lambs studied indoors and receiving tannins showed higher growth rates than those receiving tannin-free diets although the difference was not significant. Nevertheless, the limited distributed quantity of acacia (100 g per head per day) could explain the feeble benefic effect of tannins on growth. Similar results were reported suggesting that the tannin supply in low to moderate concentrations in feed can have beneficial effects for ruminants and cause a more effective use of the protein through its protection in the rumen, improving nitrogen utilization (Gladine et al., 2007; Waghorn, 2008). These results are similar to those of Ben Salem et al. (2005), who found that lambs' growth performance was improved with the complete consumption of acacia distributed at 100 g per head per day, before distributing other feeds. However, on vetch pasture, contrary to stalls, tannin-free diets foster the lambs' growth. This trend can be explained by the fact that animals in stalls were fed with hay and concentrate in which the nitrogen is in the form of protein, while for grazing animals, nitrogen is in a soluble form where the tannins cannot join nitrogen. This result agrees with those of Maamouri et al. (2011) regarding milk production improvement for sheep in stalls compared to those in pasture with the addition of tannins. Furthermore, the vetch biomass is relatively rich in tannins; the total tannin content mean values of vetch biomass averaged 360 mg CE kg^−1^ DM (Parissi et al., 2022) compared to oat hay and concentrate where tannins are not detected. The trend of dietary inclusion of tannins at low to moderate concentrations was largely documented showing that it could result in neutral or even positive effects by improving growth rate and feed utilization efficiency in ruminants, mainly due to a reduction in protein degradation in the rumen and a subsequent increase in the flow of amino acids to the small intestine (Patra and Saxena, 2011). However, the large amounts of tannin consumption may exert effects reducing feed intake, rumen microbial activity, nutrient digestibility and endogenous digestive enzyme activity, resulting in lower feed efficiency and growth rate (Huang et al., 2018; Pimentel et al., 2021).

### 4.2 Slaughter body weight, carcass weights, dressing percentages and non-carcass components

The difference in SBW among lambs is the consequence of differences in recorded ADG. Then, the difference among groups in EBW, HCW, CCW and CDP is the consequence of the lambs' different SBWs given these parameters are closely correlated to the slaughter BW (Atti et al., 2003; Hajji et al., 2016; Yagoubi et al., 2021). The CDP was higher for grazing lambs, averaging 45.8 % compared to 40.5 % for stall groups. The RDP for the P groups averaged 52.6 %, lower than previously reported results (54 %) for lighter lambs (25 kg) of the same breed (Yagoubi et al., 2021). All these parameters are affected by the feeding system but not by the acacia supply. Although grazing lambs were heavier than those studied in stalls, the gut weight was similar among groups in contrast to the results of Thériez et al. (1992), who found that some parts of the tract and particularly the rumen continue to develop as animals become heavier and older. The red organs and kidneys were more developed for grazing lambs, confirming earlier results suggesting that the physical activity affected these organs, which could be higher for animals grazing on natural plants than the stall ones (Majdoub-Mathlouthi et al., 2015; Mekki et al., 2022). However, testis weight was unchanged.

### 4.3 Carcass sectional and tissular composition

In terms of proportions in the tailed carcasses, there was no differences in carcass joints among both feeding systems; the first category of joints (leg and shoulder) averaged approximately 34 % and 20 %, respectively. These values are higher than those of Yagoubi et al. (2018, 2021) for the same breed. The loin and the breast were also more developed for lambs raised on vetch. The tail weight was higher for the P groups, averaging 950 g and 6.05 % of the carcass weight, than that of the S groups, where the tail weighed 615 g on average and represented only 4.7 % of the carcass weight. However, for both feeding systems, the tail proportion was still lower than that of Yagoubi et al. (2018), which reached 10 % for lambs of the same breed, although the lambs in the study of Yagoubi et al. (2018) were lighter. Despite this difference, the current results showing the consistency of the joint's proportion in the carcass confirm the theory of anatomic harmony reported by several works for thin-tail and fat-tail breeds (Atti et al., 2003; Obeidat et al., 2016).

All lambs deposited similar amounts of muscle, despite the different proportions of protein intake and the difference in SBW (Atti et al., 2003). Higher fat proportions were observed in the P groups. Contrary to our results, it was suggested that animals raised in stalls showed significantly higher fat percentages than those on pasture, which can be attributed to the physical activity of grazing animals (Perlo et al., 2007; Atti and Mahouachi, 2009). The tannin supply in the lambs' diet did not affect the percentages of muscle and fat, contrary to the results of Atti et al. (2003), who found that lambs whose diet contains tannins added to the polyethylene glycol (PEG) contain less fat than the animals supplemented with concentrate (20 % vs. 24.7 %).

### 4.4 Meat physico-chemical characteristics

Although pasture feeding is typically rich in fiber and poor in starch, and the acetate–propionate ratio is therefore higher, pasture-finished animals have enough glycogen to present normal ultimate pH values (Priolo et al., 2001); so, these authors, contrary to our results, suggest that the lambs studied on grass have a higher pH than those in stalls. The feeding system did not affect the ultimate pH as previously reported (Priolo et al., 2001; Majdoub-Mathlouthi et al., 2015). The initial pH for lambs that received the acacia was higher (6.9 and 6.7 for P-T and S-T, respectively) than the values of Ben Salem et al. (2005) using the same amount of acacia (6.55). The tannin consumption did not affect pH values; however, it was shown that meat from lambs fed a condensed tannin-rich diet has a higher ultimate pH compared to those receiving PEG (Bhatta et al., 2002). Conversely, it was reported that the meat pH decreased as the tannin dose increased (Liu et al., 2016). Generally, the final pH value at 24 h postmortem depends on factors such as duration of transport and handling during slaughter, age of the animal, climate, nutritional status, temperament and the health of the animal (Ponnampalam et al., 2017). However, this was not the case in the current study given all lambs were studied under similar conditions.

Water cooking loss was similar regardless of the feeding system, and the tannin supply and the acceptable pH values determine the similarity in this parameter, as the water holding capacity is strongly linked to the pH. Although no significant differences were observed, meat from animals grazing on pasture or receiving forage-rich diets tended to be darker (lower lightness values) than meat from stall-fed animals finished on concentrate-based diets, which corroborates other reported results that showed any change in meat color with feeding system. However, the meat was darker in lambs reared on grass, and the dark color can be attributed to the concentration of vitamin E in grasslands (Priolo et al., 2001; Luciano et al., 2009). Regarding the effect of the presence or absence of tannins in the diet, we observed no effect of its presence on the color. Our results are contrary to those of Priolo et al. (2002), who found the L* index to be higher for animals fed diets containing tannins, as well as other works reporting that meat issued from lambs receiving *Acacia cyanophylla* is lighter compared to those receiving a concentrate diet or receiving acacia but supplemented with PEG (Priolo et al., 2002). However, this slight difference among groups was still insignificant; the meat lightness ( L* ) values recorded for all groups averaged 43.5, indicating a light-colored meat in the range of average acceptability of meat given that a meat lightness equal to or above 34 is acceptable and is considered the value of acceptability by 95 % of consumers (Khliji et al., 2010). No differences were observed in the redness between groups receiving tannins or no tannins. However, the yellowness was slightly lower in lambs studied indoors. Luciano et al. (2009) have shown that adding tannins to a diet-based concentrate causes an increase in the value of a* ( p<0.01 ) and a decrease in the value of b* ( p<0.01 ) compared to a diet based only on concentrate. No significant differences were observed in dry matter, crude protein, ash, and fat contents among the experimental groups. Neither the feeding system nor tannin supplementation affected the overall chemical composition of the meat. This similarity may be attributed to the comparable slaughter body weights (Atti and Mahouachi, 2009), as well as to the similar nutritive value and energy levels of the experimental diets (Luciano et al., 2013).

### 4.5 Meat sensory analysis

The meat tenderness of all groups varies from 5.7 to 6, where the meat is considered moderately tender, and neither the feeding system nor the tannin supply affected this attribute. In this study, we found no differences in tenderness between animals subjected to different diets, consistent with the results of Lowe et al. (2002), who found no difference in tenderness between lambs reared on pasture and those in stalls, and others have also found that there is no direct relationship between the shear force distribution and diet (Ben Abdelmalek et al., 2019). This can be attributed to the intervention of several other factors besides diet on meat tenderness, which affirms the results of Clinquart et al. (2000), suggesting that the factors that affect the tenderness of the meat are not very dependent on farming conditions, particularly animal feeding, and the biological and technological factors are significantly larger for the tenderness of the final product (Clinquart et al., 2000). Juiciness varies from 5.1 to 5.8, meaning that the meat is moderately juicy, and there is no difference between groups. However, Priolo et al. (2002) found that meat from lambs in stalls is juicier ( P<0.01 ) compared to those on pasture. According to some authors, the fat comes from its stimulating salivary secretion; consequently an increase in lipid content of meat increases its juiciness, and thus there is a positive correlation between the fatness of the animal and the juiciness of the meat (Clinquart et al., 2000). Atti et al. (2006) have found a negative correlation between juiciness and water loss during cooking meat for a kid; it is even juicier when the percentage of water loss during cooking is lower. As the general impression varies between 5.5 and 6, the meat is perceived by the panelists as normal, and a residual flavor of fat was detected. However, Priolo et al. (2002) found that meat from animals on pasture has a pronounced liver flavor but less fat compared to lambs studied indoors ( P<0.01 ). Sañudo et al. (2000) reported that consumers not accustomed to meat from animals raised on pasture often find this product too strong in flavor, and therefore they dislike it. On the other hand, the addition of acacia in batches P1T and S1T, involves a decrease in flavor compared to other diets, while Priolo et al. (2002) found no difference in the flavor of meat issued from lambs receiving tannins and those receiving a diet free from tannins. The mean scores for all sensory attributes were above 5 on the 9-point hedonic scale, indicating that the lamb meat was generally acceptable across all treatments (Sekali et al., 2016). The flavor of sheep meat can be influenced by ultimate pH (Young et al., 2003), but this was not the case as the final pH values in the present study indicate that the development of rigor mortis was adequate during the chilling process, which results in meat with more desirable sensory properties for regular consumption and for the processing industry. The problem of pastoral flavor in sheep is mostly linked to green herbage intake, while species flavor can be linked to both grass and concentrate diets (Young et al., 2003).

## 5 Conclusions

The results of the present study demonstrate that lamb rearing on cultivated pastures improved growth performance compared to indoor feeding systems under the conditions of this experiment. The inclusion of low levels of tannins showed contrasting effects depending on the basal diet: beneficial effects on growth performance and dressing percentage were observed when tannins were supplied with a low-tannin basal diet (hay and concentrate), whereas a reduction in growth rate was recorded when tannins were added to a tannin-rich basal diet (vetch pasture). Meat physico-chemical characteristics and overall acceptability were not affected by tannin supplementation, indicating that the tested levels did not compromise meat quality. These findings highlight that the response to tannins is strongly dependent on the composition of the basal diet and that inappropriate combinations may negatively impact animal performance. Further research is needed to better define optimal inclusion levels of tannins and to establish dose–response relationships under different feeding conditions.

## Data Availability

The datasets used in this study are available upon request from the authors.
